# VPint: value propagation-based spatial interpolation

**DOI:** 10.1007/s10618-022-00843-2

**Published:** 2022-06-30

**Authors:** Laurens Arp, Mitra Baratchi, Holger Hoos

**Affiliations:** 1 Leiden Institute of Advanced Computer Science (LIACS), Leiden University, Niels Bohrweg 1, Leiden, The Netherlands; 2 University of British Columbia, Vancouver, Canada

**Keywords:** Spatial interpolation, Spatio-temporal interpolation, Missing data, Data imputation, Image inpainting

## Abstract

Given the common problem of missing data in real-world applications from various fields, such as remote sensing, ecology and meteorology, the interpolation of missing spatial and spatio-temporal data can be of tremendous value. Existing methods for spatial interpolation, most notably Gaussian processes and spatial autoregressive models, tend to suffer from (a) a trade-off between modelling local or global spatial interaction, (b) the assumption there is only one possible path between two points, and (c) the assumption of homogeneity of intermediate locations between points. Addressing these issues, we propose a value propagation-based spatial interpolation method called VPint, inspired by Markov reward processes (MRPs), and introduce two variants thereof: (i) a static discount (SD-MRP) and (ii) a data-driven weight prediction (WP-MRP) variant. Both these interpolation variants operate locally, while implicitly accounting for global spatial relationships in the entire system through recursion. We evaluated our proposed methods by comparing the mean absolute error, root mean squared error, peak signal-to-noise ratio and structural similarity of interpolated grid cells to those of 8 common baselines. Our analysis involved detailed experiments on a synthetic and two real-world datasets, as well as experiments on convergence and scalability. Empirical results demonstrate the competitive advantage of VPint on randomly missing data, where it performed better than baselines in terms of mean absolute error and structural similarity, as well as spatially clustered missing data, where it performed best on 2 out of 3 datasets.

## Introduction

Under perfect lab conditions, a data scientist can train models, infer variables of interest and discover new knowledge from neatly organised, consistent and complete datasets. However, in real-world scenarios, one is rarely so lucky. Whether it is random measurement noise, inconsistent annotation, missing data or another problem, real-world data can be messy, and tricky to process in such a way that downstream models and processes can use it effectively.

In this work, we aim to address the problem of missing data in the specific case of spatial gridded data by proposing a computational method for spatial interpolation. Prominent examples of such missing data in real-world scenarios include satellite imagery in remote sensing data (due to orbits, swaths and cloud clover Carrasco et al. 2019), mapping of ecological field measurements and samples collected at a limited set of locations (Fang et al. 2019), and local precipitation forecasting from meteorological measuring stations covering a limited set of locations (Tabios III and Salas 1985). As such, spatial interpolation is a problem highly relevant to many fields, and a large body of literature is dedicated to it in statistical domains (Heaton et al. 2019; Jiang 2018; Montero et al. 2015). Data in spatial settings is particularly susceptible to missing values, due to, among other reasons, (i) limited and/or variable spatial and temporal resolutions, (ii) limited availability of measuring locations, (iii) measurements being acquired at different times and different locations, and (iv) the characteristics of the locations in question (e.g., cloud cover or inaccessible areas). As a simplified example, consider the task of mapping the temperature at a certain time throughout the Himalayas. Since resources are limited and parts of the terrain are inaccessible, it is infeasible to collect measurements at every 100$m^2$. This gives rise to the problem of filling in the entire grid based on measurements from a limited number of locations. In this case, we could also use additional information on the elevation of the terrain to help inform our decisions – a location with a higher elevation than a reference value will likely have a lower temperature, and vice versa.

Spatial interpolation methods, such as Kriging (also known as Gaussian processes) (Jiang 2018; Cressie 2015), tend to be founded on an assumption of *autocorrelation*, meaning that values are more strongly correlated with one another the closer their spatial proximity is. Our method is no exception in this regard. However, existing methods can be categorised into *local* methods and *distance-based* methods. Local methods, such as spatial autoregressive models (Anselin 1988; Haining 1978) or convolutional neural networks (Dong et al. 2015; Shi et al. 2016), rely on adding the information of a strictly defined local neighbourhood around a target cell to enhance their predictions. The downside of these methods is that potentially valuable information outside the predefined neighbourhood is disregarded. Moreover, if local information is not available, local methods may require imputation methods to perform their estimations. Distance-based methods, on the other hand, most notably including various Gaussian process-based approaches (Jiang 2018; Cressie 2015), can use any measurement available, but rely on a distance-based weighting to use this information for their predictions. The downside of these methods is that, in most spatial settings, paths cannot be assumed to be homogeneous, and thus distance alone may not be sufficient to reliably predict values. For example, in the case of temperature measurements in the Himalayas, the difference in elevation between pairs of locations may vary despite the distance being the same. This problem is further exacerbated by the two-dimensionality of spatial problems, allowing for the existence of multiple paths between any two locations, some of which may be more important than others for the propagation of values (for example, a longer path around a mountain as opposed to a shorter path over it).

**Fig. 1:**
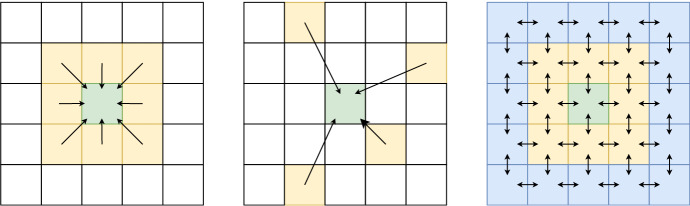
Local (left), distance-based (middle) and system-oriented (right) perspectives. In local and distance-based perspectives, the predicted value of the green cell is determined by the yellow cells (equal weights if local, unequal weights if distance-based). In the system-oriented perspective (used by our proposed method), the green cell is predicted using the yellow neighbours, which were in turn affected by their own neighbours (blue, yellow and green cells) (Color figure online)

In this work, we propose a method that incorporates a *system-oriented* perspective, illustrated in Fig. 1. In this perspective, we use a local neighbourhood to perform estimations, but we rely on recursion to propagate known values through direct neighbours over a network of (mostly indirectly) mutually interacting cells, iteratively updated until an equilibrium is reached. At every recursive call, a weight is applied to the values being propagated to represent spatial autocorrelation. This weight can furthermore be assigned dynamically in a data-driven manner, based on the features of the underlying spatial configuration. This allows for higher autocorrelation weights between, for example, two neighbouring blocks of a city, and lower weights between an industrious port and the open sea. The update rules for every cell were based on the Bellman equation for *Markov reward processes* (Bellman 1957), canonically used to estimate the value of a particular state (cell). With this perspective, we can address both the limitations of local methods and distance-based methods.

Our main contributions in this work are as follows:

- We propose a novel method, VPint, for spatial interpolation, incorporating a system-oriented perspective aimed at overcoming the limitations of existing local- or distance-based methods.
- We introduce two variants of our value propagation interpolation algorithm, both of which incorporate elements of Markov reward processes: SD-MRP, using a static discount throughout the grid and requiring no additional data, and WP-MRP, exploiting spatial features to predict neighbour-specific spatial weights.
- We provide a vectorised implementation of our methods allowing for high degrees of parallel processing to speed up the algorithm running time, which we make publicly available.^1^
- We empirically evaluate our methods on synthetic data and 2 real-world datasets and compare their performance against that of popular baselines from the Kriging, machine learning and deep learning fields in terms of mean absolute error, root mean squared error, peak signal-to-noise ratio and structural similarity. We also conducted experiments testing convergence, scalability, and whether the proposed method generalises to spatio-temporal data.

## Related work

To date, various spatial interpolation methods, both local and distance-based, have been proposed. We will discuss a selection of popular methods in this section.

*Gaussian processes* Given its widespread use, the first set of methods of note are Gaussian processes (GP), also known as Krige (1951). GPs Cressie (2015); Schabenberger and Gotway (2017) are a set of interpolation techniques based on learning the covariance of target values over distance using variogram (kernel) functions fitted to the data. Popular variants of GPs are discussed in Jiang (2018) and include ordinary Kriging (OK), universal Kriging (UK) and regression Kriging (Kriging after detrending). Contemporary contributions to GP methods include a scalable gradient-based surrogate function method (Bouhlel and Martins 2019) and a neural network-based method to overcome GPs’ limitation of disregarding the characteristics of intermediate locations in paths between pairs of locations (Sato et al. 2019). Although the assumptions made differ per variant, all GP-based methods are limited by their reliance on pair-wise distance-based covariance models. Moreover, traditional GP methods tend to scale poorly to larger datasets ($O((nm)^4)$). An overview of modern GP methods aimed at increasing the viability of GPs for large-scale datasets is given in Heaton et al. (2019), including local approximate GPs Gramacy and Apley (2015), stochastic partial differential equation approaches (Rue et al. 2017) and multi-resolution approximations (Katzfuss 2017).

*Gapfill* Gapfill Gerber et al. (2018) is a local method utilising no explanatory variables that, unlike GPs, does not build an explicit statistical model. Instead, as a local method, it relies on using subsets of the available data for its predictions. Although its local perspective and cell-specific independent predictions allow gapfill to be highly parallelised, its performance in terms of accuracy tends to fall short of GPs Heaton et al. (2019), and its dependency on the presence of sufficient amounts of non-missing values within its neighbourhood renders it infeasible for cases where missing values are clustered together.

*Belief propagation* This family of methods, particularly loopy belief propagation (Pearl 1982), has been used successfully for image denoising (Song et al. 2011), image restoration (Zheng et al. 2020) and image completion tasks (Levin et al. 2003). It generally considers graphical models (Lauritzen 1996), such as Markov random fields, and gridded datasets can also be converted to this representation. The key idea is to compute the marginal distributions of nodes in a network, based on the beliefs (estimations) of the values of the child nodes connected to them. This is done through a process of *message passing*, which iteratively propagates beliefs over the network. Conceptually, this type of method is similar to our proposed method, although it does not leverage the Bellman equation or data-driven spatial weights, and unlike belief propagation, our method computes predicted values rather than distributions thereof. While belief propagation can be considered to take the system-oriented perspective, its exact form operating on junction trees scales poorly to larger graphs ($\mathcal {O}(M \cdot N^{3})$, where *M* denotes the number of nodes and *N* is the number of discrete states per node) (McAuley and Caetano 2010) and high precision continuous variables (Zheng et al. 2012), rendering it computationally infeasible for most practical applications of grid-based interpolation tasks. Similarly, the standard approximate loopy belief propagation algorithm may have high errors compared to other methods (Satorras and Welling 2021), or even oscillate rather than converge (Murphy et al. 2013).

*Spatial regression* Spatial autoregressive models (Anselin 1988) (SAR) have remained relatively consistent, but have been expanded in some recent work (Yang and Lee 2017; Fix et al. 2021). Moving average (MA) models are often used in the context of time-series modelling (Durbin 1959), but can also be used for spatial regression problems using the “MA by AR” approach (Haining 1978). Highly related to SAR and MA models, autoregressive moving average (ARMA) models have seen recent work of particular relevance to the COVID-19 pandemic, modelling a transmission network of influenza (Qiu et al. 2021). Apart from SAR, MA, and ARMA models, which include additional features for the spatial lag and/or residuals, there are also approaches using an explicit spatial, temporal or spatio-temporal data representation, such as the tensor decomposition-based work by Corizzo et al. (2021). This latter work seems particularly relevant for spatial interpolation problems where collinearity exists within the explanatory variables. Given that the explanatory variables are being leveraged for their shared spatial structure with the target variable, collinearity in the explanatory variables is a likely scenario. However, unlike our method, these types of method are not interpolation methods, aiming instead at predicting target values directly from the spatio-temporal features or a latent representation thereof. Other recent work using spatial regression approaches include house price estimation using geographically weighted regression (Soltani et al. 2021), varying coefficient spatio-temporal regression (Lee et al. 2021), ambient black-carbon prediction (Awad et al. 2017) and an analysis of the spatial patterns of COVID-19 (Wu et al. 2021). The spatial autoregressive regression models suffer from the limitations of a local perspective: their use of a pre-defined local neighbourhood dismisses information outside of the neighbourhood radius.

*Neural networks and deep learning* Deep learning techniques, and convolutional neural networks (CNN) in particular, have been used to great effect in many computer vision applications (Dong et al. 2015; Shi et al. 2016). These computer vision-based interpolation CNNs could also be applied to general spatial interpolation. Moreover, in their 2020 publication, Hashimoto and Suto formulated a CNN architecture for the specific purpose of spatial interpolation (Hashimoto and Suto 2020). Apart from CNNs, graph neural networks (GNNs) have also been applied recently to spatio-temporal interpolation by Wu et al. (2020), utilising fully connected networks with distance-based weights determined using a random subgraph sampling strategy. Like autoregressive models, CNNs have a local perspective and therefore, dismiss potentially meaningful information outside their predefined neighbourhood. Conversely, similar to GPs, GNNs suffer from the reliance on distance-based weights, dismissing potential non-homogeneity of intermediate locations on paths between locations.

By adopting a system-oriented perspective, the method we propose in this work aims to be situated between these two main categories of existing work (local and distance-based). Moreover, like Gapfill, it offers a computational alternative to existing methods with an emphasis on explicit statistical spatial modelling.

## Problem statement

Let us define a spatial grid $\mathbf {G}$ as an $(n \times m)$ matrix, where *n* corresponds to the number of rows and *m* to the number of columns. At every cell *c* in $\mathbf {G}$, where $c = \mathbf {G}_{i,j}$, with *i* and *j* corresponding to the row and column indices in $\mathbf {G}$, respectively, there exists a true value $y^{*}_{c}$ that may be either known or unknown. If $y^{*}_{c}$ is known, we set the cell value $y_{c} = y^{*}_{c}$. If it is not known, we mark this location as unknown: $y_{c} = \varnothing$. The $(n \times m)$ matrix $\mathbf {Y}$ contains $y_c$ for all *c* in $\mathbf {G}$.

We further define a feature grid $\mathbf {X}$ as an $(n \times m \times f)$ tensor, where *f* denotes the number of features per cell. Thus $\mathbf {x}_c$ in $\mathbf {X}$ is a feature vector corresponding to location *c* in $\mathbf {G}$. We can now define a prediction model $\mathcal {M}(\mathbf {Y},\mathbf {X})$ that takes as input the available data in $\mathbf {Y}$, along with the corresponding feature vectors per location in $\mathbf {X}$, and returns a prediction matrix $\hat{\mathbf {Y}}$. The objective of spatial interpolation is to find a model $\mathcal {M}^{*}$ that minimises the mean absolute error (MAE) for all locations *c* in $\mathbf {G}$, given the predictions in $\hat{\mathbf {Y}}$. Concretely:

1 $$\begin{aligned} \mathcal {M}^{*} \in \mathop {\mathrm{argmin}}\limits _{\mathcal {M}} \sum _{c \in \mathbf {G}} |\hat{y}_c - y^{*}_{c}| \end{aligned}$$

## Methods

In this section, we will describe our proposed interpolation method in four steps. The general procedure and main philosophy will first be illustrated, after which we introduce some background for our update rules, and propose the two concrete variants of our method that we implemented. Finally, we will discuss our approach for ensuring efficient computation allowed by parallel matrix operations.

### General interpolation procedure

The core of our proposed method relies on iterative element-wise updates to an estimation grid. We first instantiate $\hat{\mathbf {Y}}$, with missing values given by $\mathbf {Y}$ being set to arbitrary real values as initial predictions (the mean of known values in our experiments). Next, for every cell $c \in \mathbf {G}$, if $\mathbf {Y}_{c}$ is known, we use it as a static prediction. If it is not known, we update its value using the *estimated* value of its neighbours $\{c' : c' \in N_S(c)\}$, where $N_S(c)$ denotes the set of spatial neighbours to cell *c*. Thus, by iterating this procedure, our algorithm recursively propagates known values throughout chains of estimated values in $\hat{\mathbf {Y}}$, through all possible paths in the system, anchored by known values.

### Background: update rule

Our update rule is based on Markov reward processes (MRPs). MRPs (Bellman 1957) are models of the form $M = \{S,T,R\}$, where *S* is a set of states $\{s_1,s_2,...,s_{|S|}\}$, $\mathbf {T}$ is an $|S| \times |S|$ matrix of transition probabilities $T_{(s,s')}$ between all pairs of states *s* and $s'$, and *R* is a set of rewards $\{r_{s_1},r_{s_2},...,r_{s_{|S|}}\}$ associated with being in a state *s*. MRPs extend Markov chains, which do not incorporate *rewards* *R*, and have been successfully used to model the behaviour of a single variable over time (Sato and Trivedi 2007; Bianchi and Presti 2016). In these temporal models, a state *s* represents a set of attribute values at a particular time *t* in a sample trajectory over time. At every *t* a state *s* can probabilistically transition from *s* to any of a set of successor states (given the current state) $S'|s = \{s'|s_{1},s'|s_{2},...,s'|s_{|S|}\}$ based on transition probabilities given by $T_{(s,s')}$, until an *absorbing state* is reached from which no further transitions are possible: $|S'|s| = 0$. Since MRPs are Markovian, the transition probability to go from *s* to $s'$ are contingent solely on *s*, and are unaffected by the history of previous states in the trajectory. If a reward $r_{s}$ is associated with the state *s*, this gives information about the desirability of state *s*. However, aside from this immediate reward $r_{s}$, intuitively the expected future rewards $\mathbf {E}(s')$ from all $s' \in S'|s$ should also be considered, as states leading to successor states with high future rewards would be more desirable. This leads to a notion of *state values*, where the rewards of all possible successor states $s'$ are used to recursively compute state values *v*(*s*) for all $s \in S$. This is typically done by iterating the Bellman equation (Bellman 1957), where the immediate reward $r_{(s,s')}$ is added to the discounted (using the discount parameter $\gamma$) average expected values of the successor states:

2 $$\begin{aligned} s': v(s) = \frac{1}{|S'|s|} \cdot \sum _{s' \in S'|s} r_{(s)} + \gamma \cdot \mathbf {E}(s') \end{aligned}$$

We opted to use this equation as our interpolation update rule. In the case of interpolation, a location *c* (at a certain time) can be seen as a state *s*, with the set of spatial neighbours $N_S(c)$ being analogous to the set of successor states $S'|s$ in MRPs. The state values *v*(*s*), then, would be the target variable $\hat{y}_{c}$ to be estimated, with immediate rewards given by known values and the discount $\gamma$ representing spatial autocorrelation. Using the Bellman equation as an update rule, we can define the set of spatial neighbours $N_{S}(c)$ as the cells $\{c' : c' \in \mathbf {G}\}$ that share a border with *c*, such that our spatial interpolation algorithm takes the form of:

3 $$\begin{aligned} \hat{y}_{c} = {\left\{ \begin{array}{ll} y_{c} &{} \text {if }\,y_{c}\,\text { known,} \\ A_S(c) &{} \text {otherwise} \end{array}\right. } \end{aligned}$$

Here, $A_S(c)$ denotes an aggregation function over the spatial neighbourhood of *c*. While in principle, it is possible to add any user-defined aggregation function, we opted to stay close to the canonical Bellman equation, by taking the mean (spatial lag) of $N_S(c)$:

4 $$\begin{aligned} A_S(c) = \frac{1}{|N_S(c)|} \,\,\, \cdot \!\! \sum _{c' \in N_{S}(c)} \gamma \cdot \hat{y}_{c'} \end{aligned}$$

Using Eq. 4 also allows us to provide an efficient vectorised implementation of our method. While the method runs for a set amount of iterations in principle, it can also incorporate an early stopping criterion by introducing a variable $\delta$, representing the change of a configuration over iterations, and using $\hat{y}_{c}^{(-1)}$ to denote the predictions from the previous iteration:

5 $$\begin{aligned} \delta = \frac{1}{m \cdot n} \cdot \sum _{c \in \mathbf {Y}} |\hat{y}_{c} - \hat{y}_{c}^{(-1)}| \end{aligned}$$

This then allows for the early stopping of the algorithm if $\delta$ drops below a user-specified threshold.

One could also consider generalising this approach to spatio-temporal interpolation problems. In that case, the algorithm cannot solely rely on Eq. 3. Whereas two spatial dimensions share the same scale, and can thus both use the same weight $\gamma$ as a spatial discount, a temporal dimension may behave very differently. As a result, to generalise to a spatio-temporal domain, we need to introduce an additional parameter $\tau$ for discounts representing temporal autocorrelation. This also leads to the set of temporal neighbours $N_{T}(c)$, which represent the same location at different time steps. Thus, the spatio-temporal update rule becomes:

6 $$\begin{aligned} {\hat{\mathbf {Y}}}_{c} = {\left\{ \begin{array}{ll} \mathbf {Y}_{c}&{} \text {if}\, \mathbf {Y}_{c}\,\text { known} \\ A_S(c) + A_T(c) &{} \text {otherwise,} \end{array}\right. } \end{aligned}$$

where $A_T(c)$ will generally use the temporal lag aggregation function:

7 $$\begin{aligned} A_T(c) = \frac{1}{|N_{T}(c)|} \,\,\, \cdot \!\! \sum _{c'_{t} \in N_{T}(c)} \!\!\! \tau \cdot \hat{\mathbf {Y}}_{c'_{t}} \end{aligned}$$

### Variants

**Fig. 2:**
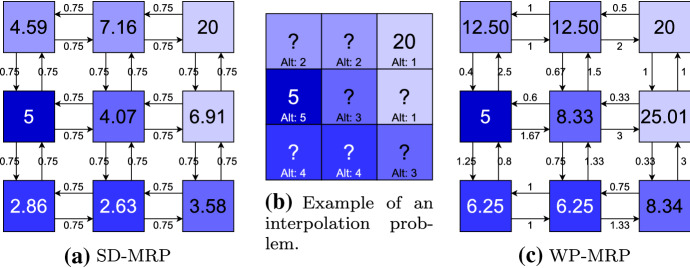
Comparison of the interpolation procedures of SD-MRP (**a**) and WP-MRP (**c**) for the example in **b**. The values in each cell represent temperature measurements, and the colour of a cell indicates the elevation of a location, where darker colours represent higher elevation. We wish to interpolate these values, such that all ‘?’ are filled with estimated values instead, based on the known values 20 and 5. In SD-MRP, a static discount of $\gamma =0.75$ was used, meaning values only decrease over distance, but do merge with one another. Meanwhile, for WP-MRP, the information on elevation was used to inform the interpolation, where in this case, the weight was chosen to be inversely proportional to the difference in features (higher elevation led to weights lower than 1 and vice versa) (Color figure online)

We propose two variants of our value propagation interpolation method. The first, SD-MRP (static discount-MRP), uses a single spatial weight parameter $\gamma$ for the entire dataset, which can be tuned using random search on subsampled data from known values. The second variant, WP-MRP (weight prediction-MRP) exploits spatial data as explanatory variables to inform its prediction of neighbour-specific weights. Unlike SD-MRP, WP-MRP would therefore not assume isotropy (the same spatial effects in all directions), although it would necessitate the use of Eq. 4 as an aggregation function. The two variants applied to the example of Fig. 2b, visualising the interpolation problem of temperature measurements in the Himalayas, are shown in Fig. 2.

#### Basic static discounts: SD-MRP

The most basic variant of our proposed method stays closest to the canonical form of the Bellman equation in Eq. 3. It uses a single discount parameter $\gamma$, ranging between 0 and 1, to represent spatial autocorrelation. This means that, for SD-MRP, values can only *decrease* over subsequent recursive calls, making known values reminiscent of a light source in the fog, radiating values around itself and merging with other light sources, but decaying over distance. In the example of spatial interpolation of temperatures, it would propagate the known temperature values over the grid, at an intensity decreasing with every recursive call, like a heat source dissipating over distance. This can be seen in Fig. 2a. The advantage of this method is that it does not require additional features to be applied to a dataset, nor does it require a prediction model to be explicitly trained. It will also regress to the initialisation value (such as 0, or the mean value) over distance, which can be a desirable property as uncertainty increases, but can also be considered a downside as it does not provide much additional information. Its main hyperparameter $\gamma$ also requires tuning, which can be done automatically by subsampling known values and performing interpolation using randomly searched $\gamma$ settings. Furthermore, the spatial characteristics of the grid are not taken into account, and isotropy is assumed. SD-MRP has a time complexity of $\mathcal {O}(4 \cdot |\mathbf {Y}| \cdot k)$, if *k* is the number of times Eq. 3 is iterated (every $c \in \mathbf {Y}$ can have at most 4 neighbours).

#### WP-MRP

In an ideal case, rather than using a single static weight $\gamma$, we would use a method allowing us to use location-specific weights $\gamma _{c',c}$. For example, when spatially interpolating temperatures in the Himalayas, knowing the difference in elevation between two neighbouring locations would enable us to know whether one value is likely to be the same, lower, or higher than the other, as illustrated in Fig. 2c. To this end, we created the weight prediction variant WP-MRP, in which we use the spatial feature vectors $\mathbf {x}_{c} \in \mathbf {X}$ and $\mathbf {x}_{c'} \in \mathbf {X}$ as inputs to a weight prediction model $\mathcal {M}_{w}$. This model predicts an individual weight of the location pair $(c,c')$ as $\gamma _{c',c} = \mathcal {M}_{w}(\mathbf {x}_{c'},\mathbf {x}_{c})$ from spatial data describing the locations (such as houses, shops and land use). $\mathcal {M}_{w}$ could consist of any machine learning model, ensemble or pipeline, but could also leverage functions directly operating on the feature space such as distance measures and inverse similarity metrics. Mapping features to a dense data manifold of lower dimensionality could also be considered.

In the case of machine learning models and pipelines, in order to train the model, we use the available cells with known true values in $\mathbf {Y}$ to supervise the training. For all pairs of neighbours $\{(c,c') : c,c' \in \mathbf {Y} \wedge y_{c} \ne \varnothing \wedge y_{c'} \ne \varnothing \}$, we would compute the true weight using the fraction $\gamma ^{*}_{c',c} = \frac{y^{*}_{c}}{y^{*}_{c'}}$, resulting in a ground truth vector $\Gamma ^{*}$ that can be used as the targets for the training of a regression model. The method for matching the elements of $\Gamma ^{*}$ to predictive features is a design choice: the location features $\mathbf {x}_{c'}$ and $\mathbf {x}_{c}$ of every location pair $(c',c)$ would need to be combined, and this could be done in any manner the situation calls for, such as adding the vectors or computing a distance metric. In our experiments we opted to simply concatenate $\mathbf {x}_{c'}$ and $\mathbf {x}_{c}$. Thus, with $\Gamma ^{*}$ and $\mathbf {x}_{c'}$, $\mathbf {x}_{c}$ for all $(c',c)$ pairs, we can train a regression model $\mathcal {M}^{*}_{w}(\mathbf {x})$, such that, if $\mathbf {Y}_{N} := \{(c',c) : (c',c \in \mathbf {Y}) \wedge (c' \in N(c) )\}$:

8 $$\begin{aligned} \mathcal {M}_{w}^{*} \in \mathop {\mathrm{argmin}}\limits _{\mathcal {M}_{w}} \frac{1}{|\Gamma ^{*}|}\,\,\, \cdot \!\!\!\!\! \sum _{(c',c) \in \mathbf {Y}_{N}} |\mathcal {M}_{w}(\mathbf {x}_{c'},\mathbf {x}_{c}) - \gamma ^{*}_{c',c}| \end{aligned}$$

Here we propose to train $\mathcal {M}_{w}$ on $\Gamma ^{*}$ using any regression (machine learning) algorithm. The full pipeline of WP-MRP using machine learning weight prediction is outlined in Algorithm 1 (which assumes available functions for model fitting). Lines 1-7 generate the elements of the true weight vector $\Gamma ^{*}$, and line 8 fits a weight prediction model to the weights found in line 4. Lines 9-22 show the iterative updates of cells in $\mathbf {Y}$, and lines 23-27 create and return the predictions in the form of a grid $\hat{\mathbf {Y}}$. The time complexity to run WP-MRP is the same as that of SD-MRP, but with the added cost of the model used for $\mathcal {M}_{w}$ (which can be chosen freely): $\mathcal {O}(4|\mathbf {Y}| \cdot k) + \mathcal {O}_{\mathcal {M}_{w}}$, if $\mathcal {O}_{\mathcal {M}_{w}}$ is the time complexity of making predictions with $\mathcal {M}_{w}$.
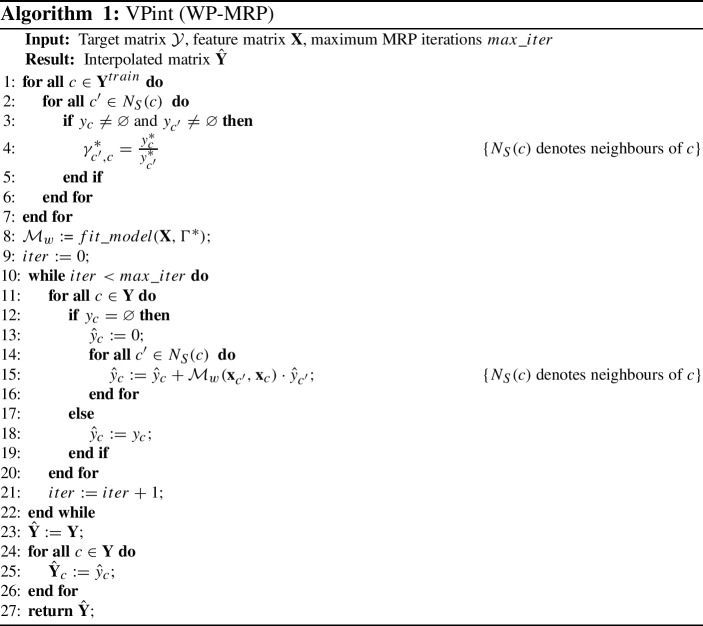


### Vector-based update rule for parallel computation

For the efficient processing of the main iterative loop of lines of our algorithms as in lines 9-22 Algorithm 1, we reformulated our update function as a series of matrix operations, allowing updates to be carried out in a highly parallelised manner through vectorisation. This approach does, however, necessitate the use of weighted averaged (spatial lag) as an aggregation function. We will illustrate the procedure on the simpler case (spatial MRP), but the approach can be generalised to spatio-temporal MRP as well. The main idea of this approach is to shuffle neighbouring values around in matrices and tensors $\mathbf {T}^{y_i}$, where the subscript *y* indicates this tensor contains values, and *i* indicates the stage of operations the data is currently in, with the accompanying neighbour weights in $\mathbf {T}^{\gamma _i}$, where $\gamma$ indicates this tensor contains weights. These operations are performed in order to compute weighted sums of neighbouring values for all cells in the grid as a matrix dot product in Eq. 11.

Concretely, let $\hat{\mathbf {Y}}$ denote a matrix of size $(n \times m)$ containing predicted values $\hat{y}_{c}$ at every cell where the true value is not known, and $y_{c}$ otherwise. We first turn this matrix into a three-dimensional tensor $\mathbf {T}^{y_0}$ of size $(n \times m \times d)$, where *d* denotes the maximum number of neighbours $max(|N_S(c)|)$ for any cell $c \in \mathbf {G}$ (in practice, this will generally be 4 as a cell can share at most 4 edges in a grid). For all *c*, the entries along the *d*-axis of $\mathbf {T}^{y_0}$ will contain the values of the neighbours of *c*. Concretely:

9 $$\begin{aligned} \mathbf {T}^{y_0}_{c,d_j} = \hat{y}_{c'} : c' \in N_{S}(c) \end{aligned}$$

If $|N_S(c)| < d$, the remaining values of the third dimension of $\mathbf {T}^{y_0}_{c}$ are set to 0. We similarly construct a tensor $\mathbf {T}^{\gamma _0}$ of size $(h \times w \times d)$, of which the entries match those of $\mathbf {T}^{y_0}$. However, the values of this tensor contain weights $\gamma _{c',c}$ from neighbour $c'$ to cell *c*, rather than the values:

10 $$\begin{aligned} \mathbf {T}^{\gamma _0}_{c,d_j} = \mathbf {\Gamma }_{c',c} : c' \in N_{S}(c) \end{aligned}$$

Next, we systematically stack all columns of $\mathbf {T}^{y_0}$ and $\mathbf {T}^{\gamma _0}$ as additional rows, resulting in the new matrices $\mathbf {T}^{y_1}$ and $\mathbf {T}^{\gamma _1}$ of size $(h \cdot w \times d)$. Now every row represents a single location *c* in a single dimension, although the information on the original columns of $\mathbf {Y}$ is kept through the order of the rows. The columns of $\mathbf {T}^{y_1}$ now show the values of the neighbouring values for a row’s location’s neighbours $N_S(c)$, and the columns of $\mathbf {T}^{\gamma _1}$ contain the corresponding weights. We now perform an MRP update by computing the dot product of $\mathbf {T}^{y_1}$ and the transpose of $(\mathbf {T}^{\gamma _1})$, and placing its diagonal values into a new vector $\mathbf {T}^{y_2}$ of size $(h \cdot w)$:

11 $$\begin{aligned} \mathbf {T}^{y_2} = diag(\mathbf {T}^{y_1} \cdot (\mathbf {T}^{\gamma _1})^{\intercal }) \end{aligned}$$

Since this vector has the same order as the rows of $\mathbf {T}^{y_1}$, we can reshape this vector into a matrix $\mathbf {T}^{y_3}$ of size $(h \times w)$, corresponding to the shape of $\hat{\mathbf {Y}}$. We now create another $(h \times w)$ matrix $\mathbf {T}^{n}$, where $\mathbf {T}^{n}_{c} = |N_S(c)|$, allowing us to divide $\mathbf {T}^{y_3} / \mathbf {T}^{n}$ element-wise, resulting in an updated prediction matrix $\hat{\mathbf {Y}}$:

12 $$\begin{aligned} \hat{\mathbf {Y}} = \frac{\mathbf {T}^{y_3}}{\mathbf {T}^{n}} \end{aligned}$$

Finally, since this operation needlessly updated known values, we substitute original known values in $\hat{\mathbf {Y}}$: $\hat{\mathbf {Y}}_{c} = \mathbf {Y}_{c}$ for all $c : \mathbf {Y}_{c} \ne \varnothing$.

Using this vectorised approach, we found that the complexity of our algorithms in terms of wall-clock time improved by a factor between 10 and 100. In order to adapt this approach to spatio-temporal MRP interpolation, which adds an extra dimension for time, $\mathbf {Y}$ is of size $(h \times w \times t)$, $\mathbf {T}^{y_0}$ and $\mathbf {T}^{\gamma _0}$ are of size $(h \times w \times t \times d)$, and *d* becomes equal to 6, as any cell can now have up to 6 neighbours. Since all neighbours are already included in the fourth dimension, there is no reason to keep the spatial and temporal dimensions separate. Thus, we can still generate the 2D matrices $\mathbf {T}^{y_1}$ and $\mathbf {T}^{\gamma _1}$, as we simply add another dimension to the stacking operation (resulting in $h\cdot w\cdot t$ rows instead of $h \cdot w$). As a result, with these exceptions, the pipeline can remain the same as it was for the spatial case.

## Experiments

In this section, we will share the details of our experiments. We will first introduce the research questions we were interested in, after which we will list the baselines we compared our method to and the datasets used in our experiments.

### Research questions

We were interested in answering the following research questions with our experiments:

- R1: How does VPint compare to baseline methods in terms of mean absolute error, root mean squared error, peak signal-to-noise ratio and structural similarity?
- R2: Does VPint converge to stable prediction values?
- R3: Can VPint be generalised to spatio-temporal problems?
- R4: Can WP-MRP leverage spatial features to perform better than SD-MRP, given sufficiently informative features?
- R5: How do VPint and baseline methods scale as the size of the dataset increases?

In addition to these main research questions, we were also interested in whether different patterns of missing data would give different results.

### Baselines

Our selection of baselines was aimed at including competitive interpolation and regression methods used for spatial and geo-spatial modelling in practice. The selection we made consists of:

- *Ordinary Kriging* (OK), using an implementation by the Python library PyKrige (Murphy 2020). Like our proposed methods, ordinary Kriging predicts values using weighted sums:
  13 $$\begin{aligned} \hat{y}_{c} = \sum _{c' \in N_{S}(c)} \gamma _{c',c} \cdot y_{c'} \end{aligned}$$
  Here $\gamma _{c',c}$ is the distance-based weight between known cell $c'$ and unknown cell *c*. However, OK uses $y_{c'}$ instead of $\hat{y}_{c'}$, $N_S(c)$ will contain more cells than only direct neighbours, and weights are determined using a distance-based variogram model.
- *Universal Kriging* (UK), also using PyKrige’s implementation. UK is highly similar to OK, but it compensates for the possible existence of a trend in the data. For both OK and UK, while more advanced methods exist, such as local approximate Gaussian processes (Gramacy and Apley 2015), as these are aimed at improving the scalability of Kriging rather than its accuracy, we consider OK and UK to be suitable representative methods for this class of algorithm.
- *Loopy belief propagation*, using a Python implementation for denoising images (Grampurohit 2021), which can be applied to interpolation problems by treating missing values as noise (generated from a uniform distribution centred around the mean of the known values, with a range based on their standard deviation). In a basic form, belief propagation is centred around the equation:
  14 $$\begin{aligned} L(\hat{y}_{c}) = \Pi _{c' \in N_S(c)} \lambda (\hat{y}_{c'}) \end{aligned}$$
  Here, $L(\hat{y}_c)$ refers to the likelihood of $y^{*}_c$ being equal to $\hat{y}_c$, and $\lambda (\hat{y}_{c'})$ is the likelihood of the neighbouring values (children) $c' \in N_S(c)$. To ultimately produce a single predicted value, the most likely value can be used:
  15 $$\begin{aligned} \hat{y}_{c} \in \mathop {\mathrm{argmax}}\limits {L(\hat{y}_{c})} \end{aligned}$$
- *Non-spatial regression*, using auto-sklearn (Feurer et al. 2019) to select the best performing regression model (or ensemble) and hyperparameter settings out of a large collection of algorithms, including linear regression, support vector regression, gradient boosted methods and others.^2^ We denote this model, which will typically be an ensemble of multiple powerful machine learning models, as $\mathcal {F}$. The resulting general form of the predictions from non-spatial regression is:
  16 $$\begin{aligned} \hat{y}_{c} = \mathcal {F}(\mathbf {x}_c) \end{aligned}$$
  We allowed auto-sklearn 150 seconds per run to find the best performing ensemble.
- *Spatial autoregressive* (SAR), *moving average* (MA) and *autoregressive moving average* (ARMA) models, using auto-sklearn to find the best performing regression model. Canonically, these models are ordinary least squares (OLS)-based linear regression methods, with extra spatial (SAR) or error (MA) terms (both in the case of ARMA). However, since we use auto-sklearn, though OLS is also a possible model, the final model will generally have a different formula, such as the potentially non-linear support vector regression models. For SAR, the spatial term is based on a spatial weight matrix $\mathbf {WM}$ and a vector $\mathbf {y}$ containing all the known values of the grid, corresponding to the rows of $\mathbf {WM}$. The general form of SAR is:
  17 $$\begin{aligned} \hat{y}_{c} = \mathcal {F}(\mathbf {x}_c,\mathbf {WM},\mathbf {y}) \end{aligned}$$
  For MA models, we used the “MA by AR” approach (Haining 1978). Its formula, using the prediction error vector $\mathbf {\epsilon }$ instead of SAR’s $\mathbf {y}$, is:
  18 $$\begin{aligned} \hat{y}_{c} = \mathcal {F}(\mathbf {x}_c,\mathbf {WM},\mathbf {\epsilon }) \end{aligned}$$
  Following the “MA by AR” approach, before we can use Eq. 18, we first needed to determine $\mathbf {\epsilon }$ using:
  19 $$\begin{aligned} \epsilon _c = y_c - \mathcal {F}_{s}(\mathbf {x}_c) \end{aligned}$$
  Here, $\mathcal {F}_{s}$ represents a separate non-spatial regression model, as in Eq. 16, to compute prediction errors on all known values. These errors can then be used by Eq. 18 by putting the values of $\epsilon _c$ for all *c* into a single vector $\mathbf {\epsilon }$. For ARMA, we again use the “MA by AR” approach for the MA component. As ARMA is a combination of SAR and MA, its formula is:
  20 $$\begin{aligned} \hat{y}_{c} = \mathcal {F}(\mathbf {x}_c,\mathbf {WM},\mathbf {y},\mathbf {\epsilon }), \end{aligned}$$
  where $\mathbf {\epsilon }$ is obtained using Eq. 19.
- *Convolutional neural networks* (CNN), optimised using automated neural architecture search (NAS). The CNN regression predicted $\hat{y}_{c}$ from $\mathbf {x}_{c}$ and $\mathbf {x}_{c'}$ for all $c' \in N_{S}(c)$, where $N_{S}(c)$ is determined by the convolutional filters of the network, similar to CNN approaches used in computer vision (Dong et al. 2015; Shi et al. 2016). We used NAS implemented by auto-keras (Jin et al. 2019) for all training sets (50 trials, 1000 epochs). Although the model architectures for CNNs can be quite complex, on an abstract level these networks are still regression models of the same form as Eq. 16.

### Datasets

Our main experiments involved a synthetic spatial dataset as well as two real-world datasets (GDP and COVID-19 trajectories), with an additional synthetic spatio-temporal dataset used to address R3. The implementation of our data generation algorithms used to create the experimental synthetic datasets is included in our public code repository; likewise, the real-world datasets are available for public use at their respective sources, allowing others to reproduce our results.

#### Synthetic data

*Spatial targets* For this synthetic dataset, based on a parameterised mean $\mu$ and standard deviation $\sigma$, the interpolation grid $\mathbf {Y}$ of user-specified size $(n \times m)$ (set to $n=50$ and $m=50$ in our experiments) was generated, where each cell *c* was assigned a base value $y^{b}_{c}$ by sampling from the normal distribution $\mathcal {N}(\mu ,\sigma )$. Next, to assign true values $y^{*}$ affected by spatial interaction, we updated every cell *c* as a weighted average (based on a *spatial autocorrelation* parameter $a^{s}$) of its own value and the mean of its neighbouring values:

21 $$\begin{aligned} y^{*}_{c} = (1-a^{s}) \cdot y^{b}_{c} + a^{s} \cdot \frac{1}{|N_{S}(c)|} \cdot \sum _{c \in N_{S}(c)} y^{b}_{c} \end{aligned}$$

*Spatio-temporal targets* To address R3, we also generated synthetic spatio-temporal data. For this type of data we introduced additional parameters for the number of timesteps *d* and the temporal autocorrelation coefficient $a^{t}$. We then built a three-dimensional tensor $\mathbf {Y}$ of size $(n \times m \times d)$ by using Eq. 21 at every time step. Since, at this point, the temporal layers of $\mathbf {Y}$ are still fully independent, we use the temporal neighbourhood function $N_{T}(c)$ to perform a final update on the cells of $\mathbf {Y}$ ensuring temporal interaction:

22 $$\begin{aligned} y^{*}_{c} = (1-a^{t}) \cdot y^{b}_{c} + a^{t} \cdot \frac{1}{|N_{T}(c)|} \cdot \sum _{c \in N_{T}(c)} y^{b}_{c} \end{aligned}$$

*Synthetic features* For our synthetic data, we created a feature vector $\mathbf {x} = (x_{c_{1}}^{b},x_{c_{2}}^{b},...,x_{c_{{|\mathbf {x}^{b}|}}})$ for every location $c \in \mathbf {Y}$. Every base feature $x^{b}_{c_{i}} \in \mathbf {x}^{b}_{c}$ was generated using a uniform distribution $\mathcal {U}(min,max)$ with user-specified *min* and *max* values. These features were then updated in a similar manner to the cell values *y*, using a parameter called the *feature correlation coefficient* *f*:

23 $$\begin{aligned} x_{c_{k}} = (1-f) \cdot x^{b}_{c_{k}} + f \cdot y^{*}_{c} \end{aligned}$$

A feature correlation coefficient *f* of 0 would result in fully random features, whereas a coefficient of 1 would result in features identical to the targets.

#### Real-world data

In the case of real-world data, the variables being measured, such as GDP or COVID-19 incidence, are generally not gridded in nature. As a result, to generate these datasets, data needs to be aggregated, e.g., by taking the mean (estimated) GDP per capita for residents in the area covered by a grid cell, or the sum of COVID-19 incidence at that location. The granularity of these datasets thus introduces a trade-off: a high granularity increases the computational cost and may result in relatively sparse datasets (as was the case in our COVID-19 dataset), but does provide a high level of detail. Meanwhile, a low granularity may result in data too low-grained to draw meaningful conclusions from, or cells that simply all regress to a global mean due to the erasure of local spatial patterns, but will be faster to compute and likely results in a higher density dataset. There is no minimal or maximum granularity cutoff point at which an interpolation method becomes infeasible. However, when gauging how applicable an interpolation method is to a users’ gridded dataset, this trade-off merits consideration.

*Gross domestic product (GDP) targets* For GDP data, we used a gridded spatial dataset containing worldwide GDP estimates sourced from World Bank (DECRG 2010) at a resolution of 1km $\times$ 1km. We specifically looked at the city of Taipei in Taiwan and its surroundings, including both heavily populated urban areas expected to have high GDP values, and surrounding sparsely populated mountainous areas with low GDP values. The resulting grid had a size of $51 \times 51$ pixels.

**Fig. 3:**
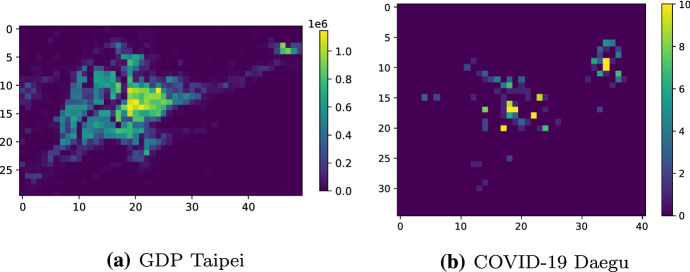
Visualisation of the GDP data in Taipei (**a**) and the COVID-19 dataset in Daegu (**b**). Due to the heavily localised large infection clusters, for the visualisation in (**b**) we limited the data to a range of [0, 10] (all values $> 10$ were set to 10) for greater visibility (the experiments used the raw values instead) (Color figure online)

*Aggregated COVID-19 trajectory targets* This dataset consisted of trajectories of confirmed COVID-19 patients prior to their diagnosis in South Korea (DACON 2020). Although this data was spatio-temporal in principle, we opted to aggregate over time both due to the relative sparsity of the data (as it was gathered at the start of the COVID-19 pandemic), and to alleviate potential privacy-related concerns in this relatively sensitive dataset. Thus, every $c \in G$ had a value corresponding to the total number of visits by people infected with COVID-19 over the entire time period. The city of interest in this dataset was Daegu, which was the main hotspot of the epidemic in South Korea at the time the data was collected. A visualisation of this data can be found in Fig. 3b. Since the target data did not come in gridded form, we set the resolution of this dataset to $35 \times 51$ pixels, putting it at a similar scale to the GDP dataset used for Taipei.

*Map-based features* To generate features for GDP and COVID-19 trajectories in South Korea and Taiwan, we aggregated a selection of vector and point map data sourced from OpenStreetMap (OpenStreetMap 2019). For all $c \in \mathbf {G}$, every element in $\mathbf {x}_{c}$ represented the count of all objects in the map data corresponding to a certain *type*, such as apartments, houses and shops. There are various design choices available for preprocessing this type of data, such as dealing with objects without an annotated type (drop or replace), feature selection (none, manually created high-level taxonomy, or keeping the most frequent types) and feature normalisation (none, unit length scaling, mean normalisation or Z-score normalisation). In accordance with the design philosophy of programming by optimisation (PbO) (Hoos 2012), we did not commit to any of these choices, and instead used a commonly used Bayesian optimisation-based automated algorithm configurator, version 0.12.0 of SMAC3 (Hutter et al. 2011), to select the best possible feature construction pipeline per method (time budget 24 hours per algorithm per dataset).

### Experimental setup

The following section will explain the procedures and experimental conditions necessary to carry out our experiments.

#### Missing data procedures

In order to evaluate our methods, we required data that was fully available to compute error metrics, while also having access to grids with missing data. To this end, we introduced two methods for ‘hiding’ known values, resulting in different patterns of missing data.

*Random missing values* This missing value approach was straightforward. Given a proportion of known values *p*, for all other cells there is a probability of being randomly obscured if a number $z = \mathcal {U}(0,1)$ sampled from a uniform distribution between 0 and 1 is smaller than *p*. That is, for every cell $c \in G$:

24 $$\begin{aligned} y_c = {\left\{ \begin{array}{ll} y^{*}_{c} &{} \text {if }\,z < p, \\ \varnothing &{} \text {otherwise} \end{array}\right. } \end{aligned}$$

In our experiments, *p* was set to 0.8.

*Spatially clustered hidden values* Much like the spatial data itself, the missing data points in a grid may not be independent, and instead subject to spatial autocorrelation themselves. For example, some locations may have missing data due to natural barriers making measurements difficult, or due to local phenomena such as clouds obscuring parts of the measurements. In this missing value approach, we were inspired by optical satellite data, where clouds are the biggest source of missing data in the field. This approach is also why algorithms like Gapfill (Gerber et al. 2018) could not be considered for our experiments, as it requires a part of the data in a neighbourhood to be available. Our method for creating clusters of missing data was based on random walks. Given a number of points *k*, a number of walks *w* and the number of steps per walk *r*, the algorithm creating artificial clusters is outlined in Algorithm 2. When applied to spatio-temporal data, the spatially missing data was applied independently to every time step.
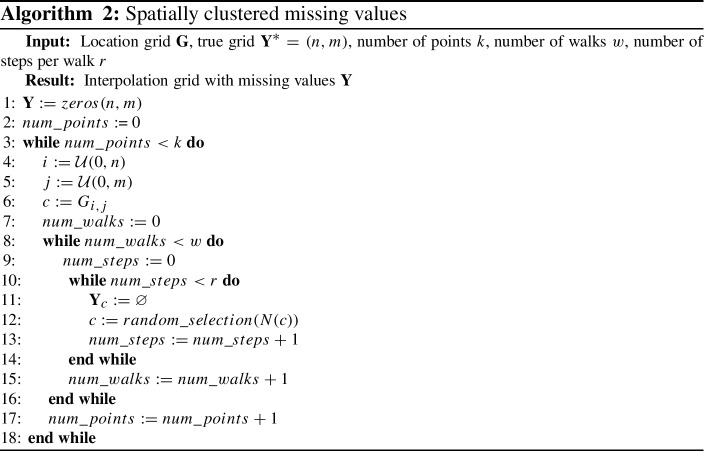


#### Experimental setup

The general form of our experiments was to run 10 algorithms (SD-MRP, WP-MRP and 8 baselines) 30 times for two types of missing data (random and spatially clustered) on every dataset (3 in total, with 1 additional dataset for spatio-temporal data), including both synthetic data and real-world datasets and addressing R1 and R3. The performance of the methods was compared according to their ranks based on the Wilcoxon signed-rank test (Wilcoxon 1992), which is similar to a t-test but does not assume normality.

For spatial synthetic data we set the size of the grid to $n=100$ and $m=100$, and for the spatio-temporal synthetic data used to address R3, we set the size to $n=50$, $m=50$ and the number of timesteps $d=5$. Tracking $\delta$ allowed us to visualise the convergence of our method (R2), using different settings for *f* in Eq. 23 allowed us to gauge the effectiveness of WP-MRP relative to SD-MRP as a function of the correlation between the features and true values of locations (R4), and varying *n* and *m* allowed us to see how well all methods scaled to larger datasets (R5). Thus, in addition to the general performance results, we used the synthetic data to run additional experiments to address research questions 2 through 5. Conversely, we used the real-world datasets to gauge how well the performance on synthetic data, and the analysis thereof, would generalise to real-world cases. For the scalability analysis we set $n=m$, with *n* ranging from 20 to 200 in steps of 20. The spatially clustered missing data was generated using $k=5$ centre points, $r = \frac{n+m}{2}$ steps per walk, and $w=\frac{r}{2}$ walks.

For every combination of a dataset with a type of missing data, all algorithms were run 30 times, and used automated algorithm configuration (for the feature preprocessing pipeline explained in Sect. 5), automated machine learning (methods using auto-sklearn, automating the selection of machine learning algorithms and their hyperparameters, as explained in Sect. 5), NAS (in the case of CNN, automating the neural network architecture explained in Sect. 5) and random search (in the case of SD-MRP’s $\gamma$, explained in Sect. 4).

All experiments were run on a computing cluster consisting of 26 homogeneous nodes containing 94 GBs of memory and using Intel Xeon E5-2683 v4 CPUs running at 2.10GHz.

## Results

In this section, we will report on the results of our experiments. We first explain the performance metrics used, after which we will cover detailed results for all individual datasets (synthetic spatial data, GDP and COVID-19). These results were computed as the mean of 30 runs per algorithm and dataset for every performance metric. After covering the dataset-specific performance metrics, we investigate other properties of VPint: qualitative visual plausibility, the convergence of Eq. 3, the degree to which it can be generalised to spatio-temporal problems, the required feature correlation for WP-MRP to perform better than SD-MRP, and the scaling of different methods to larger datasets. Finally, we will provide a high-level summary our findings.

### Performance metrics

Since multiple properties can be desirable in an interpolation method, we evaluated our method based on 4 performance metrics. The first of these was the mean absolute error (MAE):

25 $$\begin{aligned} MAE(\hat{\mathbf {Y}},\mathbf {Y^{*}}) = \frac{1}{|\mathbf {Y}|} \cdot \sum _{c \in \mathbf {Y}} |\hat{y}_{c} - y^{*}_{c}| \end{aligned}$$

MAE is the main error metric reflecting the accuracy of the predictions obtain from the methods we studied, with all errors weighted equally. We also added root mean squared error (RMSE), which penalises extreme errors relatively more severely:

26 $$\begin{aligned} RMSE(\hat{\mathbf {Y}},\mathbf {Y^{*}}) = \sqrt{\frac{1}{|\mathbf {Y}|} \cdot \sum _{c \in \mathbf {Y}} (\hat{y}_{c} - y^{*}_{c})^2} \end{aligned}$$

In addition to MAE and RMSE as basic error metrics, we also included two metrics common in the computer vision and image processing fields. The first of these is peak signal-to-noise ratio (PSNR):

27 $$\begin{aligned} PSNR(\hat{\mathbf {Y}},\mathbf {Y^{*}}) = 20 \cdot \log _{10} \frac{max(\mathbf {Y}^{*})}{RMSE} \end{aligned}$$

PSNR is highly related to the root mean squared error (RMSE) metric, and in fact contains it as a component. It computes the logarithm of the RMSE scaled by the maximal (true) value $max(\mathbf {Y}^{*})$; as such, it is expected to show similar patterns to RMSE. The main motivation for using PSNR is that it scales values by the maximal (true) value $max(\mathbf {Y}^{*})$; therefore, PSNR results will have a similar range for the GDP dataset (where errors of over $30\,000$ were common) and the COVID-19 dataset (where errors were typically under 10). Finally, we looked at the structural similarity index (SSIM):

28 $$\begin{aligned} SSIM(\hat{\mathbf {Y}},\mathbf {Y^{*}}) = \frac{(2\cdot \mu _{\hat{\mathbf {Y}}} \cdot \mu _{\mathbf {Y^{*}}}) \cdot ( 2 \cdot \sigma _{\hat{\mathbf {Y}}\mathbf {Y^{*}}} + c_{2} )}{ ( \mu _{\hat{\mathbf {Y}}}^{2} + \mu _{\mathbf {Y^{*}}}^{2} + c_{1}) \cdot ( \sigma _{\hat{\mathbf {Y}}}^{2} + \sigma _{\mathbf {Y^{*}}}^{2} + c_{2} ) } \end{aligned}$$

SSIM aims to quantify the similarity of two images in a manner consistent with human perception, emphasising spatial structure over absolute errors.

### Empirical performance (R1)

The results presented in this subsection are dedicated to answering R1. We ran detailed experiments on the synthetic spatial dataset as well as the real-world GDP per capita and COVID-19 datasets.

*Synthetic spatial data*

**Table 1:** Results for all algorithms on synthetic spatial datain terms of the average MAE, RMSE, PSNR and SSIM over 30 runs, for randomly hidden and spatially clustered hidden values

Algorithm	MAE		RMSE		PSNR		SSIM	
	Random	Clustered	Random	Clustered	Random	Clustered	Random	Clustered
Ordinary Kriging	2.392	2.59	9.004	10.446	0.386	0.379	0.06	− 0.009
Universal Kriging	2.381	2.476	9.053	9.609	0.386	0.383	0.062	0.008
Belief propagation	19.981	20.028	408.996	410.901	0.220	0.220	0.000	0.000
Non-spatial regression	2.459	2.418	9.507	9.228	0.384	0.385	0.051	0.088
SAR	2.006	2.333	6.61	8.784	0.399	0.387	**0.397**	**0.156**
MA	2.462	2.459	9.549	9.523	0.383	0.383	0.084	0.036
ARMA	1.969	**2.297**	6.357	**8.423**	0.401	**0.389**	**0.398**	**0.16**
CNN	341.277	47.391	1.263$\times 10^{3}$	35.520	0.02	0.288	0.002	− 0.0
SD-MRP	3.28	4.71	19.363	42.023	0.37	0.339	0.341	**0.156**
WP-MRP	**1.949**	2.248$\times 10^{10}$	**6.244**	1.503$\times 10^{29}$	**0.402**	− 0.272	**0.402**	0.064

All methods were ranked based on the number of other methods they significantly outperformed, established using a Wilcoxon signed-rank test ($\alpha =0.05$). The method significantly outperforming the most other methods (ties allowed) has been marked bold in every column

**Table 2:** Results for all algorithms on GDP data in terms of the average MAE, RMSE, PSNR and SSIM over 30 runs, for randomly hidden and spatially clustered hidden values

Algorithm	MAE		RMSE		PSNR		SSIM	
	Random	Clustered	Random	Clustered	Random	Clustered	Random	Clustered
Ordinary Kriging	3.863	9.375	**8.995**	31.48	− 0.514	− 0.558	0.122	0.0
Universal Kriging	3.944	9.822	**9.395**	37.977	− 0.516	− 0.568	0.121	0.0
Belief propagation	4.009	**4.048**	19.026	**21.308**	− **0.445**	− **0.437**	0.045	0.068
Non-spatial regression	8.259	9.022	27.973	32.89	− 0.563	− 0.563	0.007	0.011
SAR	6.688	7.39	20.76	28.583	− 0.55	− 0.551	0.055	0.051
MA	8.376	8.491	27.213	32.943	− 0.562	− 0.559	0.008	0.019
ARMA	6.836	6.664	21.208	24.934	− 0.55	− 0.538	0.049	0.048
CNN	5.604	8.131	30.985	45.152	− 0.568	− 0.569	0.002	− 0.001
SD-MRP	3.833	5.496	11.924	**21.121**	− 0.524	− 0.53	0.155	0.122
WP-MRP	**3.48**	1.435$\times 10^{47}$	**9.13**	2.311$\times 10^{95}$	− 0.514	− 0.945	**0.189**	**0.14**

All methods were ranked based on the number of other methods they significantly outperformed, established using a Wilcoxon signed-rank test ($\alpha =0.05$). The method significantly outperforming the most other methods (ties allowed) has been marked bold in every column

**Table 3:** Results for all algorithms on COVID-19 trajectory data in terms of the average MAE, RMSE, PSNR and SSIM over 30 runs, for randomly hidden and spatially clustered hidden values

Algorithm	MAE		RMSE		PSNR		SSIM	
	Random	Clustered	Random	Clustered	Random	Clustered	Random	Clustered
Ordinary Kriging	0.067	0.072	**1.176**	**1.368**	**0.481**	**0.555**	0.343	0.292
Universal Kriging	0.115	2.36	3.03	2936.144	0.466	**0.51**	0.452	0.273
Belief propagation	0.470	0.209	13.271	**1.335**	0.479	0.533	0.320	0.574
Non-spatial regression	0.058	0.055	**1.211**	**0.569**	**0.478**	**0.579**	0.558	0.498
SAR	0.067	0.07	**1.058**	**1.06**	**0.49**	**0.545**	0.403	0.382
MA	0.069	0.064	**1.169**	**0.936**	**0.48**	**0.571**	0.388	0.346
ARMA	0.07	0.066	1.178	**1.316**	0.481	**0.544**	0.401	0.449
CNN	0.235	883.369	7.479	6.192$\times 10^{3}$	0.407	0.182	0.796	0.005
SD-MRP	**0.036**	**0.038**	**1.175**	**1.258**	**0.481**	**0.552**	**0.941**	**0.939**
WP-MRP	0.244	0.24	8.327	7.956	0.4	0.448	0.785	0.776

All methods were ranked based on the number of other methods they significantly outperformed, established using a Wilcoxon signed-rank test ($\alpha =0.05$). The method significantly outperforming the most other methods (ties allowed) has been marked bold in every column

The results for synthetic spatial data are shown in Table 1. On this data, a fairly consistent pattern can be observed for all performance metrics: on randomly missing data WP-MRP performs best, whereas ARMA performs best on spatially clustered hidden data. It is not surprising that ARMA, as well as other regression-based methods, suffer less from missing data being clustered together since they are based on predicting values directly from features. It is more surprising that WP-MRP shows very extreme values for this type of missing data. Since SD-MRP does not suffer from the same problem, it seems that the problem lies in the weight prediction model $\mathcal {M}_w$, rather than being inherent to VPint. One possible cause for the behaviour on spatially clustered missing data may be that a mispredicted (high) weight will get disproportionately amplified with subsequent recursive calls where the target value is supposed to go up. Although these types of runs only seemed to happen on the synthetic and GDP datasets, it is a downside of WP-MRP, and one could consider constraining weights, or applying normalisation techniques, to alleviate the issue. Apart from these cases, there was no big difference between the results of randomly missing and spatially clustered missing data.

*GDP per capita* The results for GDP data are shown in Table 2. In terms of MAE and SSIM, WP-MRP was the best performing method among all methods for randomly missing data. For spatially clustered missing data, while belief propagation performed better than SD-MRP and WP-MRP suffered from extreme values hampering its performance, both VPint variants still performed well in terms of SSIM. In terms of RMSE and PSNR, belief propagation performed best in most cases, though SD-MRP performed best together with belief propagation for spatially clustered missing data, and WP-MRP, OK and UK performed better in terms of RMSE on randomly hidden data. Also, worth noting is that the performance of all methods was rather poor, with all methods achieving high error rates and low similarity scores. This may imply that it is hard to predict GDP based on spatial patterns alone (OK, UK, SD-MRP), while the map-based features were also not informative enough to make any worthwhile predictions (all other methods).

*COVID-19 trajectories* The results for COVID-19 trajectories are shown in Table 3. On this dataset, SD-MRP is performing best out of all methods in terms of MAE and SSIM, though none of the methods was clearly better in terms of RMSE and PSNR than the others in terms of statistical significance. It is, however, unfortunate to see WP-MRP as one of the two only methods performing significantly worse than all others on this dataset in these metrics, despite a high SSIM compared to baseline methods. Since other methods using feature data (apart from CNN) perform better than Kriging, it seems unlikely that the map-derived features are the cause of WP-MRP not performing well on this dataset. Instead, it appears that they are more effective for directly predicting the COVID-19 incidence at a particular location, rather than the relationship between neighbouring locations. This may be caused by the COVID-19 grid being relatively sparse; propagating values from 0 is difficult to do with a spatial weight alone. Thus, for sparse grids with mostly 0 values, SD-MRP with its decay over distance may be more appropriate, whereas WP-MRP, which can increase or decrease values based on the weights that follow from feature data, may be more appropriate in cases where all cells contain values in a non-zero range.

### Other properties (R2-R5)

We now present the results of the experiments addressing the remaining research questions, R2–R5, exploring various properties of our proposed method.

**Fig. 4:**
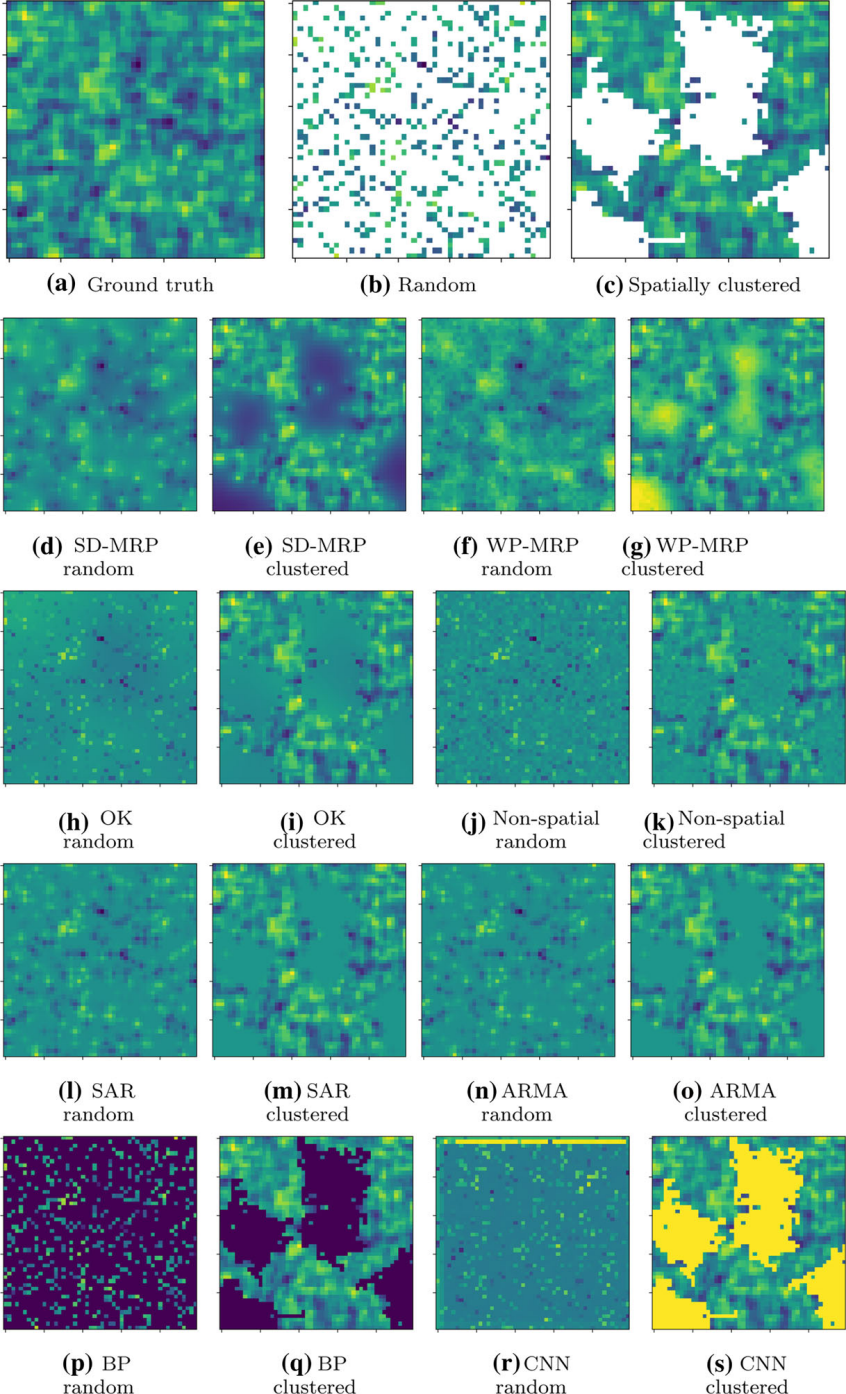
Example of synthetically generated spatial data (**a**), with random (**b**) and spatially clustered (**c**) missing data, where white pixels represent missing values in the data. Reconstructed images by SD-MRP (**d**, **e**), WP-MRP (**f**, **g**), ordinary Kriging (**h**, **i**), non-spatial regression (**j**, **k**), SAR (**l**, **m**), ARMA (**n**, **o**), belief propagation (**p**, **q**) and CNN (**r**, **s**) are shown in the lower rows of the figure. The results for universal Kriging and MA were highly similar to those of ordinary Kriging and ARMA, respectively, and are not shown here (Color figure online)

**Fig. 5:**
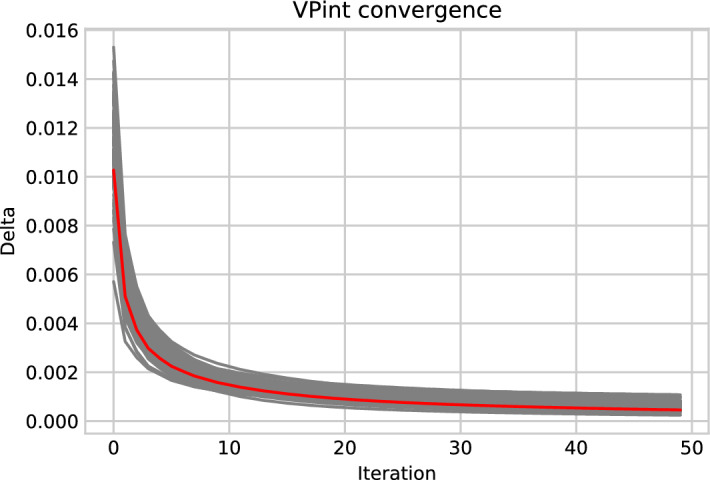
Convergence of WP-MRP over 100 runs on randomly hidden synthetic spatial data. The y-axis shows $\delta$, or the amount of change from the configuration from one iteration to the next as a proportion of the mean value of the prediction grid. All individual runs were plotted in grey, with the mean $\delta$ values plotted in red. The algorithm showed stable convergence with low variability over 50 iterations (Color figure online)

*Visual plausibility* An example of hidden synthetic data ($n=m=50$) is shown in Fig. 4, with a visual comparison between its reconstruction by the different methods. The reconstructed images caution against relying too much on mean absolute error, as all methods (apart from belief propagation and CNN) were able to reach a similar mean absolute error as WP-MRP or better (around 2.2 for random, 0.9 for clustered). However, our methods (and WP-MRP in particular) appear to capture the spatial characteristics of the original image, captured by the structural similarity index ($SSIM=0.34$ for random, $SSIM=0.69$ for spatially clustered), better than OK (which seemingly simply predicts the average value, $SSIM=0.04$ for random, $SSIM=0.65$ for spatially clustered). Compared to non-spatial regression, our method delivers less noisy interpolations, while also blurring less than ARMA. The results for belief propagation and CNN catch particular attention, as in these cases belief propagation vastly underestimated the values, whereas CNN overestimated them orders of magnitude higher than other methods. The latter implies that there may be a large risk of overfitting for the neural networks, due to the models being too complex for the limited amount of training data available.

*Algorithm convergence (R2)*

An example of the convergence of WP-MRP over iterations can be seen in Fig. 5. As the figure shows, as the algorithm iterates Eq. 3, it converges to a stable configuration which we use for our predictions. Moreover, the variability of this convergence was fairly low, indicating that the running time of the algorithm will be relatively stable regardless of the situation. This example considered the convergence of WP-MRP on randomly hidden synthetic spatial data, but similar behaviour could be observed for SD-MRP, and on different datasets with spatially clustered hidden data. This includes the convergence of WP-MRP on spatially clustered hidden data for synthetic spatial data, where Table 1 earlier indicated that WP-MRP did not perform well.

*Generalising to spatio-temporal problems (R3)*

Addressing R3, to gauge whether our method could also be applied to 3-dimensional spatio-temporal problems, we ran an additional set of experiments on synthetic spatio-temporal data. The results of this experiment are shown in Table 4. Unfortunately, it appears that our proposed method does not (yet) generalise well to 3-dimensional problems, as both VPint variants were the worst performing out of all methods. However, other modifications adapting VPint to the spatio-temporal domain may be more successful. Interestingly, on this synthetic dataset, ARMA performed best across the board – one might have expected a more inherently spatio-temporal method, like Kriging, would have performed better. Given this, it may be the case that the spatio-temporal version of VPint performs badly not due to an inherent problem with the method, but rather a lack of exploitable patterns in the temporal dimension of the data. Whereas Kriging methods will tend to give lower weights to non-informative variables, in its current form, our method weights all dimensions equally, meaning that a non-informative dimension would harm the performance of our method rather than helping it. In the synthetic data we used in our experiments, independent spatial data was generated for all time steps individually, after which temporal autocorrelation was simulated using Eq. 22. As every time step had two essentially independent neighbours, the autocorrelation between the two may have cancelled out one another on some cells, leading to a diminished performance. As a result, the model may perform better on real-world spatio-temporal data under favourable conditions. However, as this is outside the scope of this work focused on spatial interpolation, further research would be necessary to establish exactly what those favourable conditions would be.

**Fig. 6:**
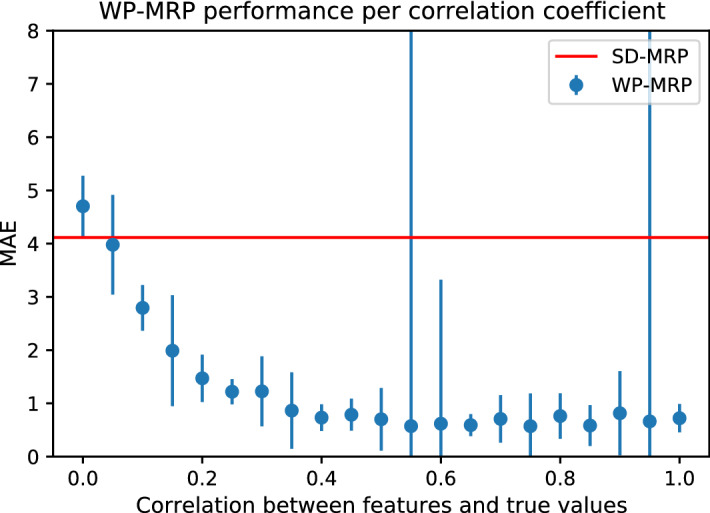
SD-MRP and WP-MRP performance on synthetic data for various settings of *f*. All datapoints were computed using the median and standard deviation of 30 runs per setting (SD-MRP is unaffected by features, and therefore constant). The extreme error bars at $f=0.55$ and $f=0.95$ also show the effect of WP-MRP producing extreme values (Color figure online)

**Table 4:** Results for all algorithms on synthetic spatio-temporal data in terms of the average MAE, RMSE, PSNR and SSIM over 30 runs, for randomly hidden and spatially clustered hidden values

Algorithm	MAE		RMSE		PSNR		SSIM	
	Random	Clustered	Random	Clustered	Random	Clustered	Random	Clustered
Ordinary Kriging	2.741	3.12	12.025	15.714	0.373	0.362	0.001	0.011
Universal Kriging	2.67	3.118	11.465	15.824	0.375	0.361	0.008	0.002
Belief propagation	3.994	4.012	408.672	412.397	0.220	0.220	0.000	0.000
Non-spatial regression	2.516	2.538	9.871	10.039	0.382	0.381	0.001	0.001
SAR	2.635	2.451	11.369	9.588	0.376	0.383	0.046	0.069
MA	2.466	2.524	9.663	10.025	0.383	0.381	0.001	0.001
ARMA	**2.021**	**2.257**	**6.668**	**8.292**	**0.399**	**0.389**	**0.412**	**0.193**
CNN	3.245	4.293	16.646	204.488	0.364	0.344	0.0	0.0
SD-MRP	15.931	14.648	269.688	241.627	0.239	0.249	0.042	0.038
WP-MRP	7.332	8.296	81.403	117.111	0.29	0.275	0.046	0.047

All methods were ranked based on the number of other methods they significantly outperformed, established using a Wilcoxon signed-rank test ($\alpha =0.05$). The method significantly outperforming the most other methods (ties allowed) has been marked bold in every column

*Performance of WP-MRP compared to SD-MRP as a function of feature correlation (R4)* To address R4, we ran an additional experiment on synthetic data with settings of the feature correlation coefficient *f* in Eq. 23 ranging between 0.05 and 1.0 in steps of 0.05. Figure 6 compares the performance of the two methods based on MAE as a function of feature correlations. The figure shows the error distribution acquired from 30 runs. As expected, Fig. 6 shows that WP-MRP performs better than SD-MRP for high values of *f*, and conversely, SD-MRP appears more successful for low feature-target correlations. However, there appear to be diminishing returns for higher *f* after 0.4, and already at a correlation of 0.1 WP-MRP performed better than SD-MRP on the synthetic data. In conclusion for R4, this experiment shows that WP-MRP leverages spatial features to perform better than SD-MRP in situations where the features are sufficiently informative.

**Fig. 7:**
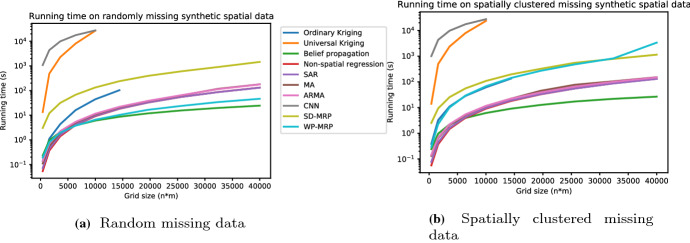
Running time in seconds of the full pipeline of different methods as a function of grid size on synthetic spatial data. The results for universal Kriging and CNN were cut off early due to hitting time-out thresholds. Meanwhile, although the running time of ordinary Kriging was not too different from other methods, its memory usage became prohibitively large and exceeded its allotted resources (13 GB) (Color figure online)

*Scaling to larger datasets (R5) * Addressing R5, we ran a scalability analysis by running every algorithm once on synthetic data for grid sizes ranging from 20 to 200 (height and width) in steps of 20. The results of these experiments, based on the total running time of methods (including training, if any, but excluding NAS, SMAC and other algorithm configuration as they are optional) can be seen in Fig. 7a (random) and b (spatially clustered). The figures show that SD-MRP, while faster than CNNs, does not scale well to larger datasets, and that WP-MRP scales similarly compared to non-spatial regression, SAR, MA and ARMA. This tells us that the iterative MRP-derived update rule likely does not account for a large portion of the running time; instead, it appears that the auto-sklearn training procedure, much like in the case of non-spatial regression and SAR, MA and ARMA is the main bottleneck for WP-MRP. The reason, then, for SD-MRP to scale poorly, would be the random search-based subsampling procedure used to find an optimal static discount $\gamma$ explained in Sect. 5.

We can also see in both figures that universal Kriging scales very poorly to larger datasets; in fact, its runs timed out after grids of the size $100 \times 100$. While CNN was slightly less affected than UK by the increasing size, its running times were still exceedingly high, and likewise hit a time-out threshold after $100 \times 100$ grids. Similarly, while the running times of ordinary Kriging were similar to those of other methods, its memory usage became prohibitively large by exceeding its allotted 13GB at $120 \times 120$ grids. Thus, this experiment showed another weakness of GPs, namely their high memory usage, which is also detrimental to their scalability. Newer GP methods, like local approximation GPs (Gramacy and Apley 2015), may scale better in terms of running time and memory usage by using local approximations, although this may come at the expense of a decreased ability to capture global information.

In conclusion for R5, our methods scale better than Kriging to larger datasets, on par with non-spatial regression, SAR, MA, and ARMA, though SD-MRP did take longer than these methods on randomly missing data. Generally, our methods use substantially less memory than ordinary Kriging and universal Kriging.

### High-level summary

Tables 1, 2, 3 show the competitive advantage of the VPint variants, in terms of MAE and SSIM. For randomly missing data, the two VPint variants together performed better in terms of MAE than baseline methods on all 3 spatial datasets, although individually both methods only performed better than all baselines on 2 out of 3 datasets. WP-MRP performed better than all other methods on synthetic and GDP data, though SD-MRP also performed better than baseline methods on the GDP data, and SD-MRP performed better than all other methods on the COVID-19 dataset. In terms of SSIM, the two VPint variants together were again the best performing methods on all 3 datasets, where WP-MRP again performed best on the synthetic (though tied with SAR and ARMA, with SD-MRP following one ranking lower) and GDP datasets, and SD-MRP also performed better than the baseline methods on GDP data. SD-MRP performed better on the COVID-19 dataset, where WP-MRP was the third best performing method after CNNs. In terms of RMSE, the results were less consistent, with no method clearly outperforming the others across all datasets, and as expected, the rankings for PSNR and RMSE were almost always the same. This difference implies that, while the VPint variants will often perform better *on average*, when they do fail to produce good results, the errors will be more extreme than those of baseline methods. This was seen especially clearly in runs where WP-MRP obtained error values orders of magnitude higher than all other methods.

On spatially clustered missing data, in terms of MAE, SD-MRP still performed best on the COVID-19 dataset, but was outperformed by belief propagation on GDP data and by ARMA on synthetic data. WP-MRP also failed to significantly outperform baseline methods on any of the datasets for this type of missing data. However, in terms of SSIM, both VPint variants performed significantly better than all baselines on GDP and COVID-19 data, and SD-MRP tied with SAR and ARMA for synthetic data. Since it seems that SD-MRP preserves the spatial structure of spatially clustered hidden data better than baseline methods on all 3 datasets, and WP-MRP did so on 2 out of 3 datasets, we conclude that VPint would be a better option for this type of missing data if the spatial structure of the interpolations is important. Interestingly, SSIM is higher for all methods for spatially clustered data; this is likely caused by this type of missing data being considered a substantial structural element, thus affecting SSIM less than random missing data.

Regarding our additional experiments, we found that the convergence of VPint tends to progress smoothly and has very little variance between runs (as seen in Fig. 5). Table 4 shows that, in its current form, our method does not yet generalise well from the spatial case to the spatio-temporal case. Figure 6 shows that WP-MRP will perform better than SD-MRP starting from a feature correlation coefficient *f* of around 0.1 for synthetic data, implying that a high correlation between features and targets is not required for the feature data to have added value to the method. Finally, Fig. 7 showed favourable scalability of our proposed method, particularly compared to Kriging.

## Conclusion and future work

In this work we proposed VPint, a value propagation-based method for spatial interpolation, establishing a system-oriented perspective. To this end, we introduced two variants of our interpolation method (SD-MRP and WP-MRP), the latter of which exploits spatial features describing the characteristics of the grid. In our experiments, VPint was found to perform significantly better than baseline methods in terms of mean absolute error and structural similarity on randomly missing data in 3 datasets, and 2 out of 3 datasets for spatially clustered missing data. Overall, whether VPint is the appropriate choice of algorithm appears to depend on the type of data in question, and the goals of the user. In the common case where a low error rate is the objective, particularly in a way that preserves the spatial structure of a grid, VPint (and especially WP-MRP) will generally be the best option for randomly missing values. On spatially clustered missing data, SD-MRP is usually a better option, though other methods are more competitive on this type of missing data. Despite these advantages offered by VPint, if the user is looking for a method that does not suffer from outliers of particularly bad predictions, and is willing to accept higher average errors as a result, other methods may be a better option.

In future work, it would be interesting to focus on exploring the performance of our methods on other real-world datasets, particularly when using other sets of features not derived from map data. Furthermore, we see value in further analysis of the spatio-temporal variant of VPint, focussed on the circumstances under which it will perform well. Alternatively, a different approach to spatio-temporal interpolation could be a temporally layered version of WP-MRP, using a representation similar to the tensor-based approach adopted by Corizzo et al. (2021). Such an approach would eliminate the need for explicit feature data, and would instead use known values at different time steps as features to derive spatial weights. This type of approach may well be worth exploring. Finally, applying this method to specific fields frequently suffering from missing data, such as cloud cover in remote sensing data, may contribute greatly to those fields. The need for cloud removal techniques, given that about 70% of Earth is covered by clouds at any time, is great; existing techniques are limited and often either difficult to apply for non-experts in deep learning, or fail to produce actionable new information (such as simply predicting a mean, or replacing cloudy pixels). A data-driven, universally and easily applicable method able to fill in cloud cover (or gaps caused by faulty sensors), informed by the highly correlated features of previous imagery at the same place, could increase the availability of data and therefore the efficacy of the many high-impact methods dependent thereon to a large extent.

## Data Availability

The code used to generate synthetic data is included in the project’s code repository. All real-world data used is publicly available: the GDP dataset can be found at https://datacatalog.worldbank.org/search/dataset/0037850, and the COVID-19 dataset can be found at https://www.dacon.io/competitions/official/235590/data/.

## Footnotes

1 https://github.com/ADA-research/VPint.

2 Auto-sklearn is an automated machine learning (AutoML) package that allows automatic algorithm selection, hyperparameter optimisation and feature preprocessing ensuring that a high-performing pipeline is selected on given dataset.

## Code Availability

The code used for the experiments is publicly available at https://github.com/ADA-research/VPint.
